# The magnetization transfer ratio of the post-mortem canine intervertebral disk is positively correlated to Pfirrmann grading on high field 3.0T MRI: a pilot study

**DOI:** 10.3389/fvets.2024.1335331

**Published:** 2024-02-14

**Authors:** Antonin Martenne-Duplan, Corentin Tricou, Marlène Finck, Benjamin Cartiaux, Germain Arribarat, Giovanni Mogicato

**Affiliations:** ^1^Centre Hospitalier Vétérinaire Massilia, Animedis, Marseille, France; ^2^Anatomy and Diagnostic Imaging Department of Ecole Nationale Vétérinaire de Toulouse (ENVT), Université de Toulouse, Toulouse, France; ^3^Toulouse Neuroimaging Center (ToNIC), University of Toulouse Paul Sabatier-INSERM-ENVT, Toulouse, France; ^4^Toulouse Neuroimaging Center (ToNIC), University of Toulouse Paul Sabatier-INSERM, Toulouse, France

**Keywords:** dog, MRI, nucleus pulposus, degeneration, MTR

## Abstract

**Objective:**

Intervertebral disk (IVD) degeneration usually occurs earlier in chondrodystrophic dog breeds than in other breeds. Spinal cord compression secondary to IVD degeneration is the most common cause of myelopathy in these dogs. Standard magnetic resonance imaging (MRI) sequences permit the identification of IVD degeneration and its consequences on adjacent neurological structures. In human medicine, quantitative MRI sequences, such as magnetization transfer ratio (MTR) sequences, are developed and used to detect early IVD degeneration. This prospective randomized post-mortem comparative study aimed to evaluate the correlation between a qualitative Pfirrmann MRI grading and the MTR values of the IVD in chondrodystrophic dogs.

**Materials and methods:**

Vertebral columns of eight canine cadavers were frozen and thawed prior to imaging with T2-weighted and MTR sequences using a 3.0 T high-field MRI. These sequences were reviewed by two observers. A Spearman correlation coefficient was calculated in order to compare the MTR values with the Pfirrmann grade. Pearson correlation coefficients were calculated to evaluate the inter-observer agreement of the delineation of the region of interest (ROI) around the NP and the MTR values. A Wilcoxon-Mann–Whitney test was used to conclude on the significance of the correlation between the MTR values and the Pfirrmann grades.

**Results:**

There were 138 intervertebral disks analyzed: 29/138 (21.0%) IVD were grade I, 74/138 (53.6%) grade II, and 35/138 (25.4%) grade III. No grades IV and V were present in this study. Inter-observer agreement for delineation of IVD ROI was fair (*r* = 0.54) but inter-observer agreement of mean MTR value within the ROI was very good (*r* = 0.89). Mean MTR values were 16.459% (10.0305–21.0950%) for grade I, 18.888% (10.0750–27.2400%) for grade II, and 22.813% (12.5700–31.7600%) for grade III. The mean MTR value was significantly different between each Pfirrmann grade: between grades I and II (*p* < 0.005), grades II and III (*p* < 0.05), and grades I and III (*p* < 0.005). There was a significant moderate positive correlation between Pfirrmann grading and mean MTR values (*r* = 0.516).

**Conclusion:**

The magnetization transfer ratio seems to be an objective method to detect early intervertebral disk degeneration via quantitative analysis.

## 1 Introduction

The intervertebral disk (IVD) is composed of an incompressible nucleus pulposus (NP) surrounded by an annulous fibrosus (AF), and by the vertebral endplates on its cranial and caudal aspects (1). The role of the IVD, with the ligamentous structures, is to limit vertebral column motion in any plane and to resist compressive forces along the long axis of the vertebral column that are created by the weight of the head and the abdominal muscles (2).

IVD degeneration is a common disorder in dogs (3). It occurs by two main pathophysiologic mechanisms, chondroid and fibroid degenerations, also named Hansen type 1 and Hansen type 2, respectively, (2). IVD degeneration is a common finding in clinically neurological normal dogs, especially in chondrodystrophic breeds with frequent and early chondroid degeneration of the NP. In veterinary medicine, magnetic resonance imaging (MRI) is the gold standard for evaluating IVD degeneration and spinal cord consequences due to its excellent tissular contrast in the case of intervertebral disk herniation (4). A 5-category Pfirrmann system is used in human medicine to classify lumbar IVD degeneration. This scoring takes into account the intensity of the signal and the height of the IVD. This scoring has been previously used in veterinary medicine and has shown a great correlation to histopathological degeneration (4). However, this grading is a qualitative method with limitations especially concerning inter-operator agreement.

In human medicine, MRI quantitative IVD evaluation has been developed using quantitative MRI sequences such as magnetization transfer ratio (MTR). MTR sequence provides a measurement of the rate of magnetization exchange between the free hydrogen protons in fluid and those that are bound to macromolecules in tissue and is highly predictive of histopathological changes (5). The MT is assessed by applying an off-resonance RF pulse at a different frequency than the Larmor frequency of free-water protons. This RF only saturates the protons bound to macromolecules, such as collagen. Collagen has been found to produce a significant magnetization transfer effect (6). Collagen is the main macromolecule of IVD AF. Collagen is also present in the physiological NP in lower proportion. This macromolecule is produced by chondrocyte-like cells during chondroid IVD degeneration of the NP. A previous study using a canine model showed that early degenerative or traumatic IVD changes are detectable with both T2WI and MTR MR imaging and are well correlated to histopathological findings (7). This same study showed that MTR increases with the severity of T2WI IVD signal abnormalities and IVD degeneration degree on histopathology. Another human study showed a moderate and significant linear correlation between MTR and Pfirrmann grades suggesting that collagen relative density increases with the degeneration of the NP. It showed that MTR MRI may serve as a non-invasive diagnostic tool for IVD degeneration (5). However, to our knowledge, IVD degeneration grade evaluation using MTR has never been attempted in veterinary research.

So the aim of this study was to evaluate the correlation between the Pfirrmann intervertebral disk degeneration grading on T2WI images with the quantitative magnetization transfer ratio values of post-mortem intervertebral disks on a 3.0 T high-field MRI. It also aimed to evaluate the inter-observer agreement of the delineation of the region of interest (ROI) around the NP and the MTR values.

Our first hypothesis was that quantitative MTR would be significantly positively correlated to Pfirrmann grading. The second hypothesis was that MTR values have a high inter-observer agreement.

## 2 Materials and methods

This study was a prospective randomized post-mortem comparative study. The experimental protocol and euthanasia conditions were approved by the Ethical Committee ‘Sciences et Santé Animale 115 - Ecole Nationale Vétérinaire de Toulouse with the authorization number APAFIS# 21559-2019071917392588v3. The dogs were raised in the Avogadro Laboratory (Fontenilles, France) and were euthanized for pedagogic purposes unrelated to this study at the Ecole Nationale Vétérinaire de Toulouse (ENVT) in the Anatomy Department. Cervical and thoracic vertebral columns were harvested from the cadavers and frozen at −20°C for approximately 5 months. Four days before the MRI acquisition, vertebral columns were placed in a cold room at 4°C to achieve slow defrosting. Once completely thawed, the vertebral columns were stored at 4°C. On the day of the MRI, vertebral columns were stored in a cooler and transported from the Pavillon Baudot of the Center Hospitalier Universitaire de Purpan (Toulouse, France) to perform the acquisition.

### 2.1 Image acquisition

MR Imaging was performed using a high field 3.0 Tesla magnet (Achieva, Philips, Amsterdam, The Netherlands). For imaging, the vertebral column was placed with the cervical part facing the MR gantry and the ventral aspect of the vertebral bodies facing the patient table to mimic a “sternal recumbency’-like position. “Dorsal recumbency”-like position was not achievable due to instability of the vertebral column on the table during acquisition. A 16-channel flexible elbow coil (Philips) was placed around the vertebral column. The field of view included the cervical and thoracic spine. T2-weighted images were obtained in the sagittal plane with the detailed MR acquisition parameters displayed in Table 1. Then a magnetization transfer (MT) sequence was performed in the sagittal plane using the same placement of slices as the T2-weighted sequence with detailed acquisition parameters also displayed in Table 1. The MTR data were obtained using a sagittal gradient echo sequence with dual acquisition and collected with or without the application of a magnetization transfer pre-pulses. One acquisition was performed with an off-resonance pulse applied at 1000 Hz below the free water proton resonance frequency for 2.5 ms (MSAT) and the second one without the off-resonance pulse (M0). Then, a third set of images consisting of the Magnetization Transfer Ratio images was calculated on a pixel-by-pixel algorithm using the formula: MTR = (M0-MSAT)/M0. The signal value of MTR can be expressed as the ratio value (0–1) or as the percentage of saturation (0–100%) calculated with the formula: MTR (%) = [(M0-MSAT)/M0]x100. In the result section below, the authors decided to express the results as the percentage of saturation.

**Table 1:** Image acquisition parameters for the T2-weighted images and for the magnetization transfer ratio (MTR) sequence.

Sequence	T2WI sagittal	MTR sagittal (M0)	MTR sagittal (MSAT)
Repetition time TR (ms)	3,000	72	72
Echo time TE (ms)	90	4, 5	4, 5
Angle flip (°)	18°	18°	18°
Matrix	400*400*16	400*400*16	400*400*16
Voxel size (mm)	0,7*0,7	0,7*0,7	0,7*0,7
Slice thickness (mm)	2	2	2
Interslice gap (mm)	0	0	0
Number of slices	16	16	16
Echo train (slice)	1	1	1
Acquisition number	3	3	3
Acquisition time (min)	3	27	27
Band width (Hz)	1,200	1,200	1,200
Pre-saturation impulsion RF	-	-	1,000 Hz for 2.5 ms

### 2.2 Qualitative analysis - Pfirrmann grading

T2-weighted images were reviewed by a first-year ECVDI veterinary radiology resident (AMD) and a ECVDI-certified veterinary radiologist (MF) using a Digital Imaging and Communication in Medicine (DICOM)-reader system (3.0, Horos Project, Geneva, Switzerland) in a high-resolution screen workstation (iMac, Apple, USA). To grade each IVD space, the image was adjusted to display the highest height and length of the NP hyperintensity. The image used for grading could be different in the same vertebral column depending on the IVD of interest. The observers graded the IVD by consensus using the 5-category Pfirrmann system to grade the degree of IVD degeneration. Due to the post-mortem nature of this study, the comparison between NP and cerebrospinal fluid signals was not evaluable for the grading. The IVD was considered grade I if the disc was homogeneous with bright hyperintense signal and normal disc height; grade II if the disc was inhomogeneous but with preserved hyperintense signal, clear differentiation between the NP and the AF and normal height; grade III if the disk was inhomogeneous with intermittent gray signal intensity, unclear differentiation between the NP and the AF and normal to slightly decreased height; grade IV if the disk was inhomogeneous with hypointense dark gray signal intensity, absence of distinction between the NP and the AF and a slightly to moderately decreased size and grade V if the disk was inhomogeneous with hypointense black signal intensity, absence of distinction between the NP and the AF and a collapsed disk space. Both observers were familiar with the Pfirrmann grading procedure and had trained together prior to this study on various human and dog T2WI images to homogenize their grading.

### 2.3 Quantitative analysis - magnetization transfer ratio

For the comparative study, ROI was drawn separately by a first-year ECVDI veterinary radiology resident (AMD) and a final-year veterinary student (CT) using a DICOM reading system (MRIcron, 2007, USA) for each IVD. The ROI was drawn over the hyperintense signal of the NP in the T2WI images (See figure in Supplementary materials file). Each ROI was saved and then transferred to another software (Sisyphe, France) using the “copy/paste” function on the corresponding image of the MTR sequence (8, 9). For each ROI, the following data were recorded: ROI volume, minimal MTR value (MTRmin), mean MTR value (MTRmean), median MTR value (MTRmed), and maximal MTR value (MTRmax). For the comparative study, for each ROI, the average value of both operators’ MTRmean was used (mMTR). Then, for all the IVD in the same Pfirrmann grade, an average of all the IVD values was calculated (mean MTR).

### 2.4 Comparative study - statistical analysis

Results were assessed by using commercially available softwares: Excel 2018 (16.16.27 version, Microsoft Corporation, USA), R 2004–2016 (3.6.3 version, R Core Team, New Zealand) and Prism 10 (10.1.1 version, GraphPad Software, USA). Pearson’s correlation coefficients were used to assess the inter-operator agreement for the ROIs volume and the MTR mean of the nucleus pulposus. The agreement strength was classified as very poor (<0.19), poor (0.2–0.39), fair (0.4–0.59), good (0.6–0.79), and very good (>0.8). A Spearman correlation coefficient was also calculated in order to compare the MTR values with the Pfirrmann grade. The significance threshold was set to value of *p*<0.05 for all statistical sets.

In addition, box plots of MTR values as a function of Pfirrmann grade were produced using the Excel software (16.16.27 version, Microsoft Corporation, USA). The average value of the different MTR for each grade is represented by a black cross within the box.

Finally, to conclude on the significance of the correlation between the MTR values and the Pfirrmann grades, a Wilcoxon-Mann–Whitney test was used.

## 3 Results

Eight vertebral columns were included in this study. All dogs were beagle, considered as a chondrodystrophic breed. Dogs had a median age of 2 years and 7 months (range 23–36 months) and a median weight of 14.4 kg (range 12.8–16.0 kg) at the time of euthanasia. Five were neutered males and three were spayed females. Dogs presented no abnormalities on clinical and neurological examinations. The number of IVD visible on the MRI sequences was variable for each dog. A total of 138 IVD were analyzed in this study (range: 15–19 IVD per dog).

### 3.1 Pfirrmann grading and MTR sequences

After T2WI assessment, 29/138 IVD were grade I (21.01%), 74/138 grade II (53.62%), and 35/138 were grade III (25.36%). No grades IV and V were present in this study.

**Figure 1:**
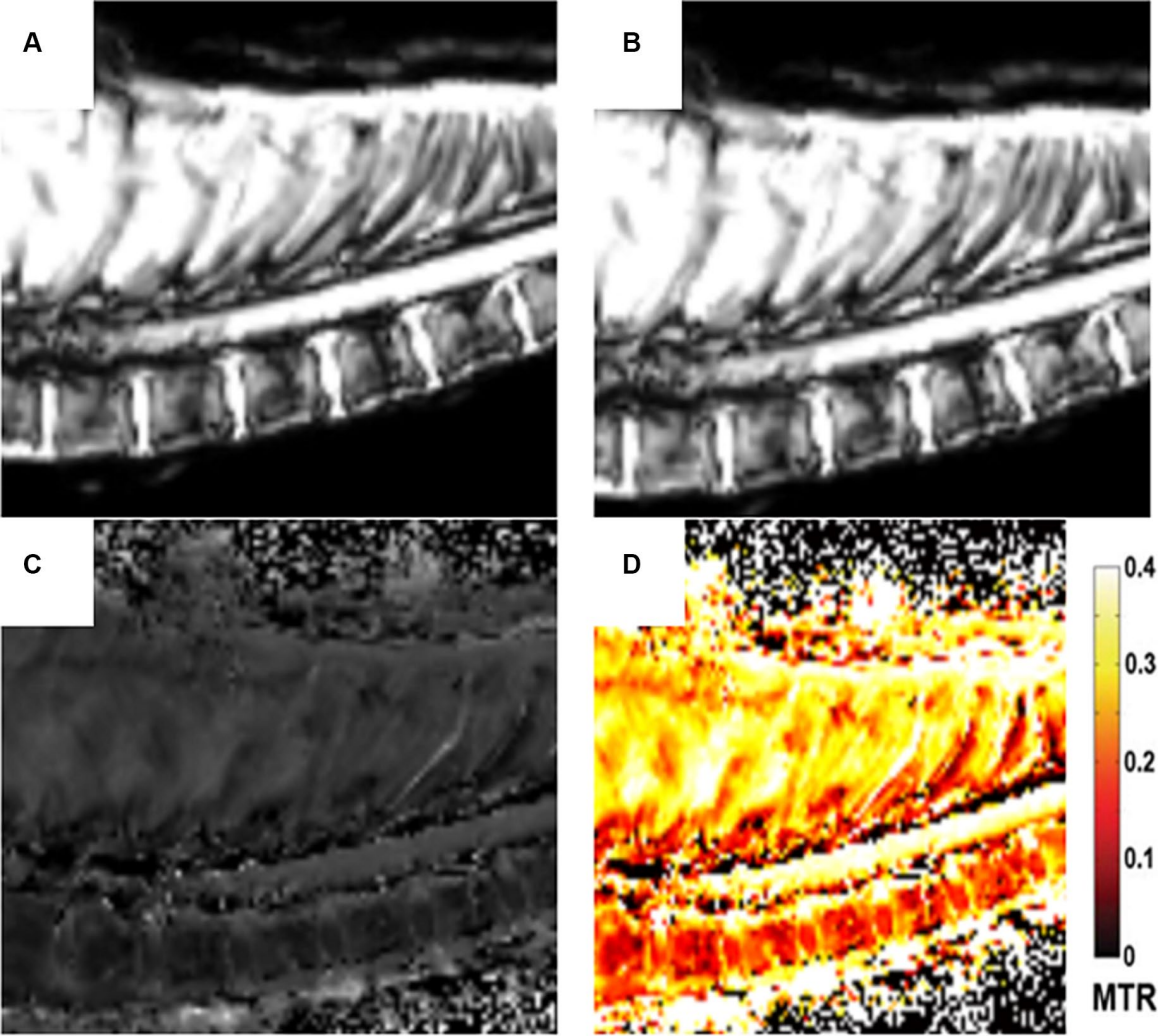
Different sets of images of the MTR sequence: Image without the off-resonance RF with the signal M0 **(A)**. Image with the off-resonance saturation RF with the signal MSAT **(B)**, Image calculated with the MTR pixel-by-pixel algorithm (MTR = (M0-MSAT)/M0) **(C)**. Image of the MTR color map **(D)**.

Four types of images were obtained from the magnetization transfer sequences: image without the off-resonance RF with the signal M0, image with the off-resonance saturation RF with the signal MSAT, image calculated with the MTR pixel-by-pixel algorithm (MTR = (M0-MSAT)/M0) and image of the MTR color map. These images are displayed in Figure 1.

**Figure 2:**
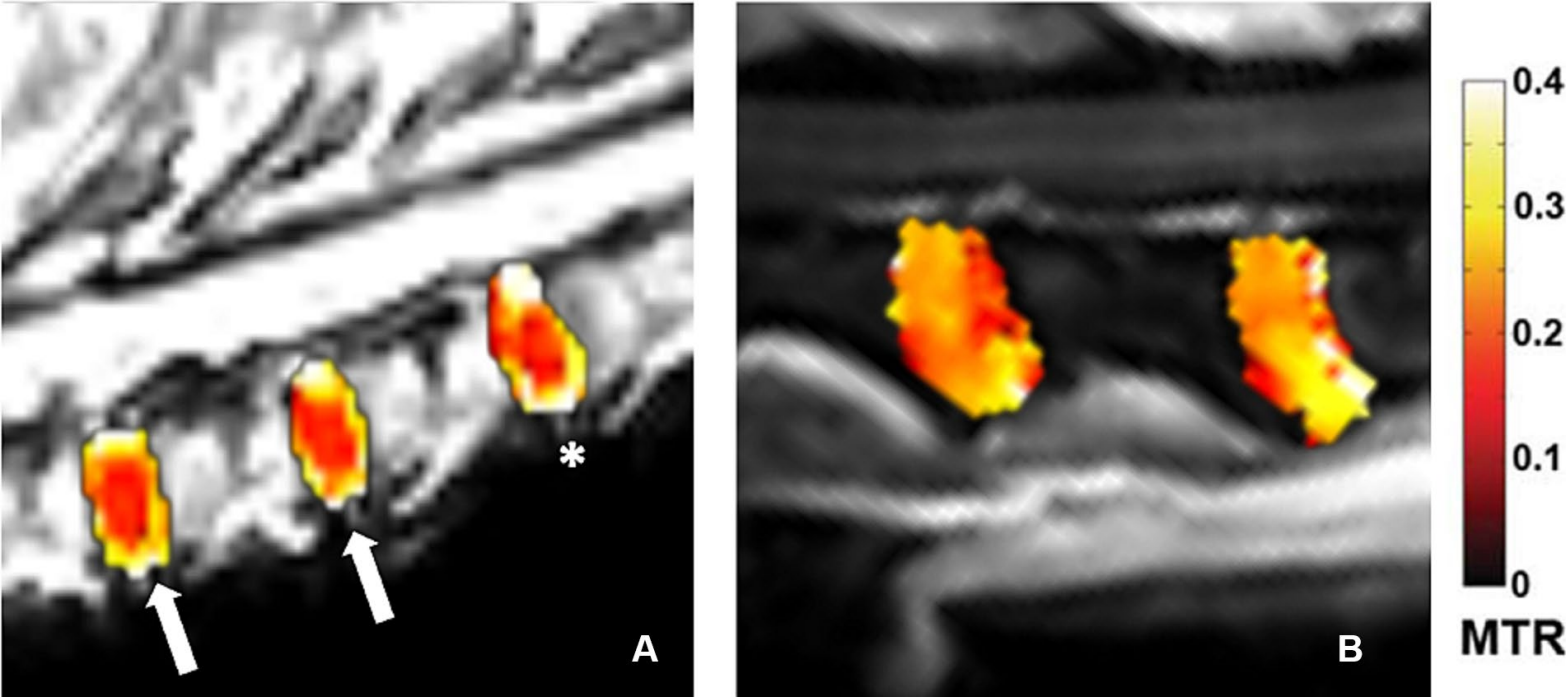
Overlaid MTR Color maps ROI of intervertebral disks. **(A)** – white arrows: grade I, the NP appears as a homogeneous low-saturated signal (red), and around the NP the well-defined annulus fibrosus of mildly increased saturation (yellow). **(A)** – asterisk: grade II, heterogeneity of the NP and the annulus fibrosus is less well demarcated than in grade I. **(B)**: grade III, the NP appears moderately heterogeneous with increased saturation and the annulus fibrosus is barely visible.

A greater signal saturation in the MTR sequences was observed with increased Pfirrmann grade: the mean MTR value for Pfirrmann grade I IVD degeneration was 16.459% (10.0305–21.0950%), the mean MTR value for Pfirrmann grade II IVD degeneration was 18.888% (10.0750–27.2400%), and the mean MTR value for Pfirrmann grade III IVD degeneration was 22.813% (12.5700–31.7600%). The overlaid MTR color maps (Figure 2) offer a clear visualization of the NP. In grade I (Figure 2A white arrays), the NP appears as a homogeneous low-saturated signal (red), and around the NP the well-defined annulus fibrosus of mildly increased saturation (yellow). In grade II (Figure 2A asterisk), the findings were variable with heterogeneity of the NP and/or increased saturation of the NP center, and the annulus fibrosus is less well demarcated than in grade I. In grade III (Figure 2B), the NP appears moderately heterogeneous with increased saturation and the annulus fibrosus is barely visible.

### 3.2 Statistical analysis

**Figure 3:**
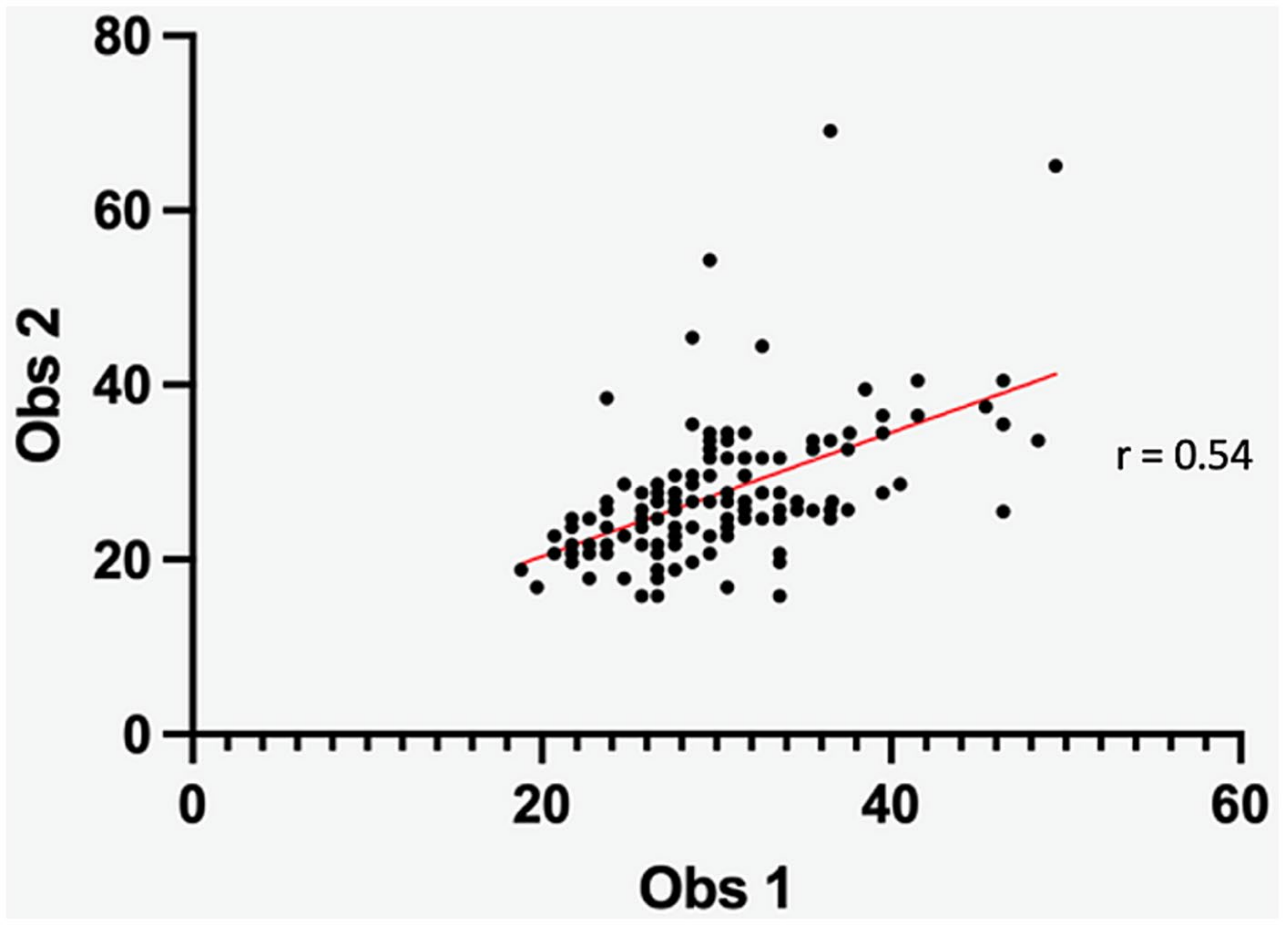
Scatter diagram depicting the delineation of IVD ROI between observers. The Pearson correlation coefficient (0.54) indicates a fair inter-observer difference.

**Figure 4:**
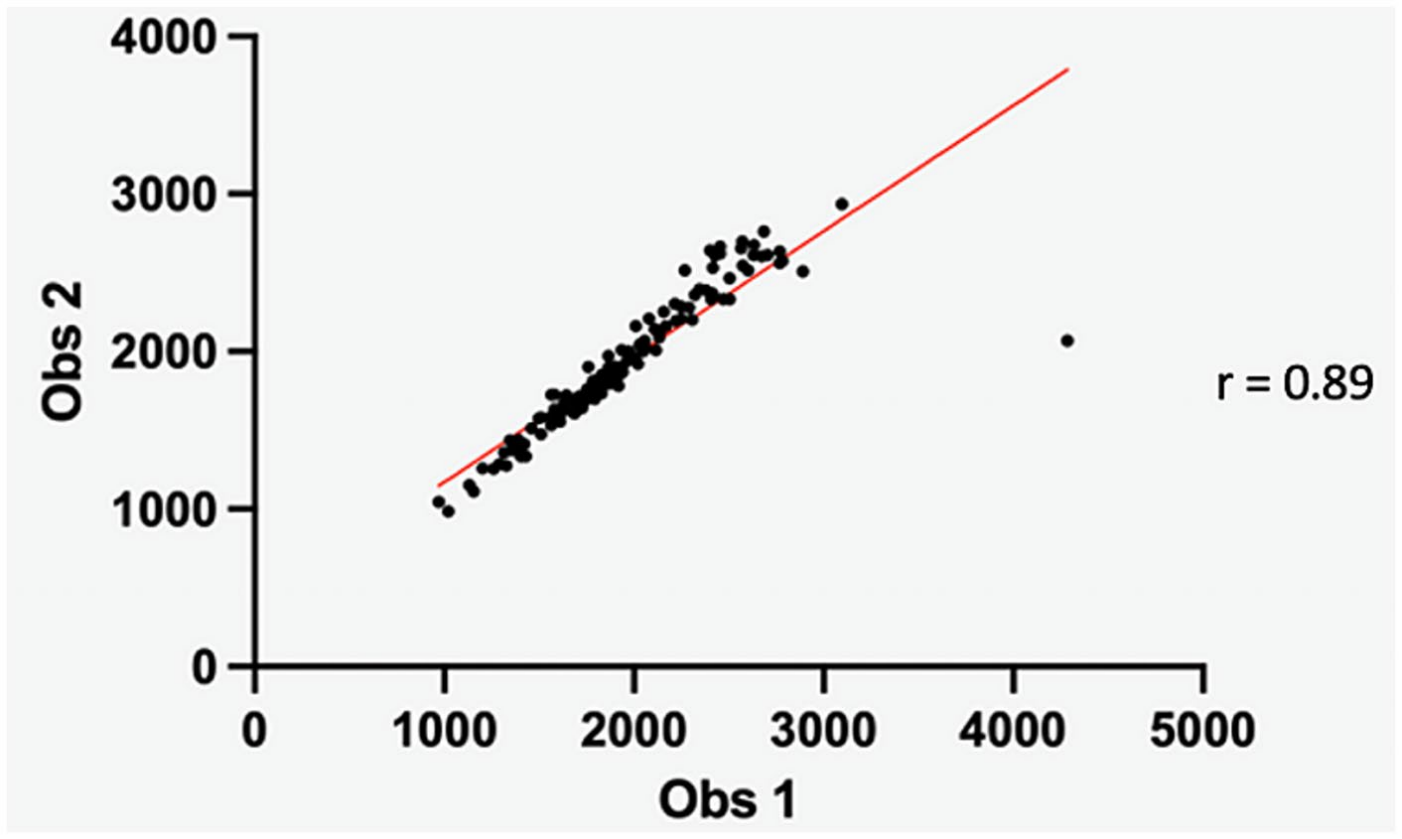
Scatter diagram illustrating the inter-observer MTR mean of the nucleus pulposus. The Pearson correlation coefficient (0.89) indicates a very good inter-observer agreement.

The Pearson’s correlation coefficients for the inter-operator agreement for the measurement of ROIs volume and the MTR mean of the nucleus pulposus were fair (0.54) and very good (0.89), respectively (Figures 3, 4).

**Figure 5:**
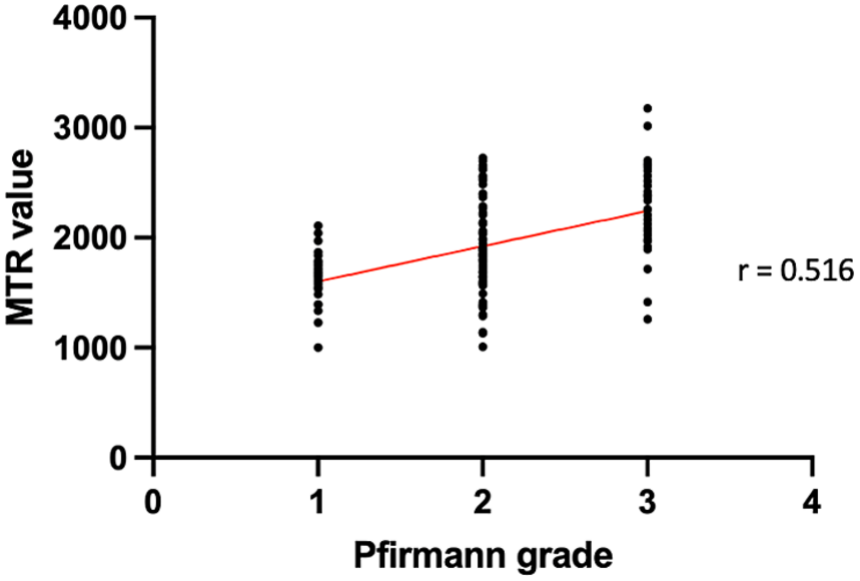
Distribution of MTR values according to Pfirmann grades. The Spearman correlation revealed a statistically significant positive moderate correlation (rho = 0.516; *p* < 0.001) between Pfirrmann grades and MTR values of the IVD.

The Spearman correlation showed a significant positive moderate correlation (rho = 0.516; *p* < 0.001) between the Pfirrmann grades and the MTR values of the IVD (Figure 5).

**Figure 6:**
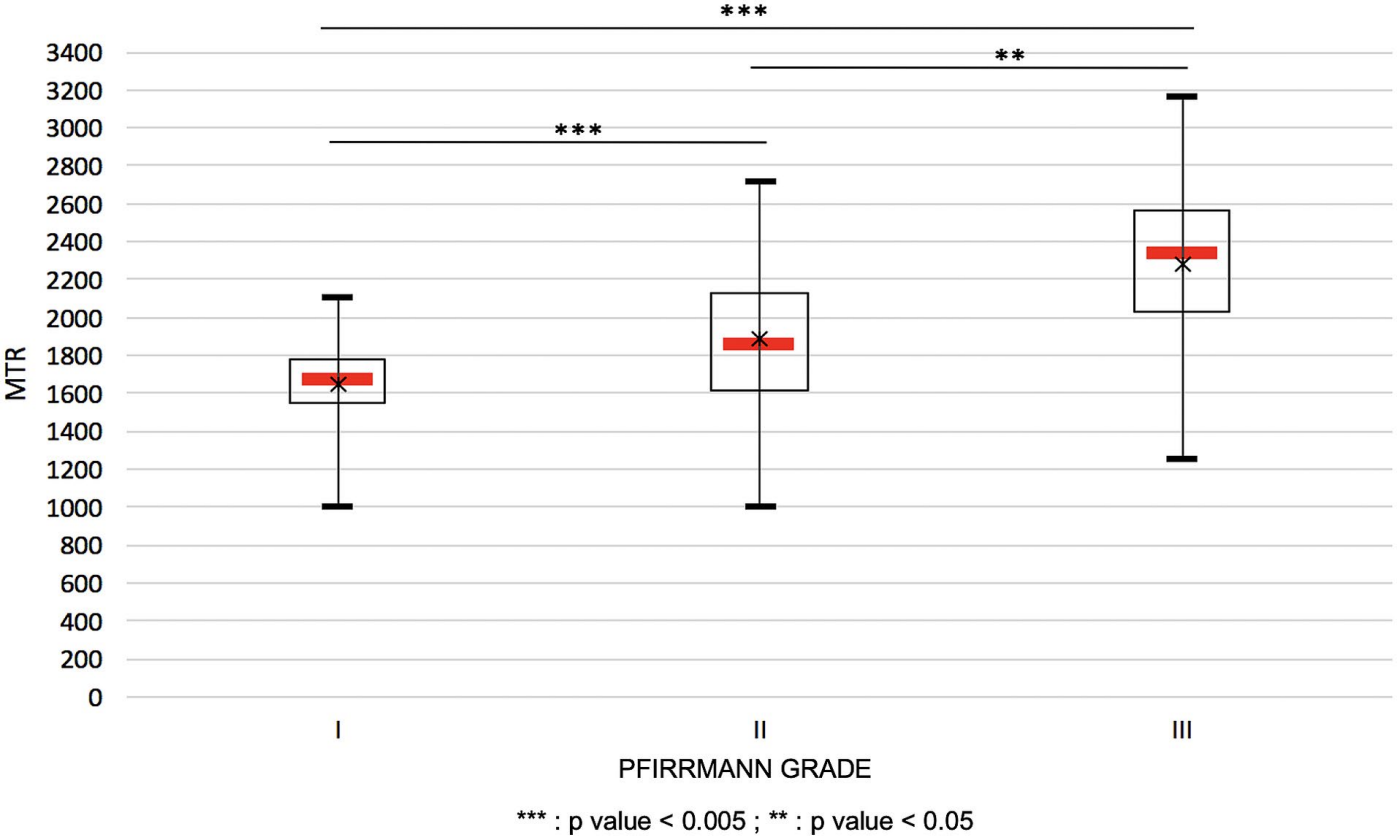
Variation of the MTR values in function of the Pfirrmann grading represented in plot boxes. The black cross represents the average of MTR within one Pfirrmann grade.

The statistical analysis showed that there were significant differences between all the Pfirrmann grades and their correlated MTR values: between grades I and II (*p* < 0.005), grades II and III (*p* < 0.05), and grades I and III (*p* < 0.005). These results are depicted in box plots in Figure 6.

## 4 Discussion

The standard assessment for IVD degeneration is based on T2-weighted images of the NP and also on the Pfirrmann grading depending on the radiologist’s preference. The Pfirrmann grading only relies on qualitative observations such as the signal intensity and the shape of the NP in T2-weighted images (4, 10). This is prone to intrapersonal and interpersonal biases and as the Pfirrmann grading is a numerological grading, some IVD degeneration may share features of two adjacent grades. In contrast, the MTR images are based on quantitative measurement with specificity of certain macromolecules content, providing a more objective assessment than the previously mentioned methods (11). Therefore, this study evaluated correlations between the IVD NP MTR values and Pfirrmann grade in dogs.

In human studies, quantitative MRI techniques have been employed to identify IVD degeneration by linking MRI signals with the biochemical components of the NP and AF. Superior to semi-quantitative morphological MRI methods like traditional T2-weighted imaging, quantitative MRI techniques, particularly MTR imaging, exhibited heightened accuracy (12–14). A recent study with 67 human patients affected by IVD degeneration at the cervicothoracic junction revealed a positive correlation between MTR values of the IVD NP and Pfirmann grades (15). In our study, we observed similar results with comparable MTR values.

IVD degeneration stands out as one of the most prevalent neurologic disorders in dogs (3). Traditional diagnostic measures for IVD degeneration encompass clinical signs, neurological manifestations, and imaging observations. While various diagnostic imaging techniques exist, MRI holds the status of the preferred method for delineating the stage of disc degeneration, the extent of herniation, and concurrent spinal cord parenchymal lesions in dogs (16–18). Conventional MRI protocols in canines typically involve two-dimensional (2D) fast spin-echo (FSE) sequences replicated across multiple planes, and more recently, three-dimensional (3D) FSE (19). However, quantitative MRI techniques, especially Magnetization Transfer Ratio (MTR) imaging, remain underutilized. A recent study has shown modifications of the spinal cord MTR values secondary to IVD herniation with significantly decreased MTR values in chronic cases compared to acute cases (20). Another experiment using a canine model demonstrated the detectability of early traumatic or degenerative changes of the IVD through MTR, confirmed by histologic and biochemical analysis (7). Nevertheless, as of now, no study has assessed the application of MTR imaging in correlation with Pfirrmann grading for IVD degeneration.

In our study, the MTR values within the ROI showed a strong inter-operator agreement despite a low inter-operator agreement for the ROI volume. IVD of grade I appear in MTR images as a homogeneous low-saturated signal with a well-defined border of mildly increased saturation. The higher saturation of the ventral and dorsal aspect of the NP might be secondary to volume averaging on T2-weighted images leading to inclusion within the ROI of the inner part of the AF or of the transitional zone with a higher collagen content than the NP resulting in higher saturation. In grade III, the NP appears moderately heterogeneous with increased saturation. The technique used to draw the ROI in our study has already been described in previous studies (21, 22) and the strong inter-observer agreement of the MTR values within the ROI in our study showed that the method of ROI drawing may be a reliable method.

Hansen’s study published approximately 70 years ago described the pathological features of IVD degeneration in dogs and classified it into two distinct forms each typically occurring in different types of dogs (2). Despite general support of Hansen’s original description, more recent histologic analysis suggested that there is less difference between degeneration in chondrodystrophic and non-chondrodystrophic dogs than was previously assumed. In fact, advanced AF degeneration in non-chondrodystrophic dogs has been shown to be associated with the replacement of notochord-like cells by chondrocyte-like cells similar to IVD degeneration in chondrodystrophic dogs (23, 24). In our study only the MTR values of the MP were analyzed with a ROI drawn over the hyperintense signal of the NP in the T2WI images. So it would be interesting to study the MTR values of the AF. Morevover only Beagle dogs, considered as a chondrodystrophic breed, were imaged. The collagen content of the IVD has been shown to vary with the breed and more specifically with chondrodystrophic status (25). A further study is necessary to know if our results might be transposable to non-chondrodystrophic dogs as well.

The Pfirrmann grading system focuses on IVD characteristic changes such as signal intensity, disk structure, differentiation between AF and NP, and IVD height but not on changes in the tissues surrounding the IVD. These changes in the surrounding tissues should be included to obtain a complete picture of the status of IVD. In fact, to accurately evaluate the IVD degeneration in dogs, the Pfirrmann grading system should be used in conjunction with the presence or absence of disk herniation and abnormalities of the other structures composing the IVD space (17). To obtain this information, transverse and midsagittal MRI images are needed. In our study, this modified Pfirrmann wasn’t usable because of the absence of CSF due to the transection of the central nervous system at the level of the foramen magnum.

Several limitations are acknowledged with this study design. The first main limitation of this study is the frozen post-mortem *ex vivo* protocol. In human literature, the freezing process appears to be a good method of tissue conservation when the sample is rapidly imaged after defrosting. This technique showed minimal alteration of the image quality in MRI (26). In other species, it has been shown that frozen–thawed samples have a significantly increased MTR compared to lived samples, however the alteration of the MTR signal was homogeneous in the entire scan field of view (27). In our study, all the vertebral columns were homogeneously frozen and thawed. For this reason, difference of MTR between different Pfirrmann grades in our samples was considered interpretable in frozen–thawed cadavers. One limitation of this study is the restriction to the cervical and thoracic areas. In fact, a human study showed that thoracic IVD NP had a higher collagen content compared to the lumbar IVD NP, resulting in higher MT saturation in the thoracic region than in the lumbar region (5, 28). Another limitation of this study is the absence of advanced IVD degeneration of grades IV and V using the Pfirrmann grading. Further studies of the entire *in vivo* vertebral column with severely degenerated IVD are necessary to confirm our results in live animals.

Finally, one of the main clinical limitations of this study is the acquisition time of the MTR sequence of 27 min. This acquisition time seems non-compatible with daily clinical use and may only be feasible in clinically stable animals in whom significant prolongation of anesthesia time would not result in severely increased anesthetic risk. Furthermore, only sagittal images were acquired in our study but transverse images may provide new diagnostic information and lengthen even more the anesthetic time.

In conclusion, we statistically demonstrated a significant moderate positive correlation between Pfirrmann grading and the MTR values, showing that there is an increased magnetization transfer within the NP with IVD degeneration. The MTR MRI seems to be an objective method to detect early IVD degeneration via quantitative analysis. Nonetheless, additional investigations are required to validate our findings in canine patients from both chondrodystrophic and non-chondrodystrophic breeds.

## Data availability statement

The original contributions presented in the study are included in the article/Supplementary material, further inquiries can be directed to the corresponding author.

## Ethics statement

The animal study was approved by Sciences et Santé Animale 115 - Ecole Nationale Vétérinaire de Toulouse with the authorization number APAFIS# 21559-2019071917392588v3. The study was conducted in accordance with the local legislation and institutional requirements.

## Author contributions

AM-D: Conceptualization, Investigation, Methodology, Writing – original draft. CT: Conceptualization, Formal analysis, Validation, Writing – review & editing. MF: Conceptualization, Investigation, Methodology, Supervision, Validation, Writing – review & editing. BC: Conceptualization, Investigation, Supervision, Validation, Writing – review & editing. GA: Conceptualization, Methodology, Supervision, Validation, Writing – review & editing. GM: Conceptualization, Funding acquisition, Investigation, Methodology, Supervision, Validation, Writing – review & editing.
