# Supplementary material for: The magnetization transfer ratio of the post-mortem canine intervertebral disk is positively correlated to Pfirrmann grading on high field 3.0T MRI: a pilot study
Source: Front Vet Sci. 2024 Feb 14;11:1335331. doi: 10.3389/fvets.2024.1335331 (PMC10899331; doi:10.3389/fvets.2024.1335331)
Supplement: Supplementary file 1 [file Data_Sheet_1.DOCX]

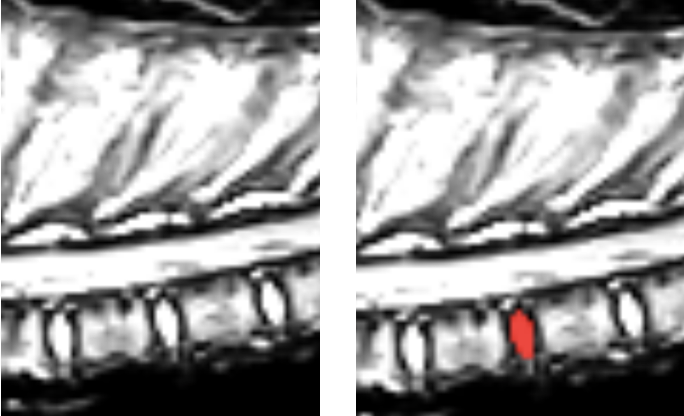


Supplementary materials : Drawing of the ROIs focused on the nucleus pulposus in hypersignal in T2-weighted imaging.
